# Cognitive signs and symptoms in people with a psychiatric diagnosis on semaglutide: a retrospective cohort study of 13 007 patients in the USA

**DOI:** 10.1136/bmjment-2026-302828

**Published:** 2026-08-20

**Authors:** Riccardo De Giorgi, Nadia Lipunova, Elgan Mathias, Fei Shang, Maxime Taquet

**Affiliations:** 1 Department of Psychiatry, University of Oxford, Oxford, England, UK; 2 Oxford Health NHS Foundation Trust, Oxford, England, UK; 3 Department of Experimental Medicine, University of Salento, Lecce, Italy; 4 Holmusk Technologies Inc. UK, London, UK

**Keywords:** Bipolar and Related Disorders, Depressive Disorder, Mental Health, Psychopharmacology, Schizophrenia Spectrum and Other Psychotic Disorders

## Abstract

**Background:**

Cognitive deficits across psychiatric disorders are common and difficult to treat. Semaglutide, a GLP-1 receptor agonist, shows potential cognitive benefits in diabetes and obesity, but effects in psychiatric populations remain unclear.

**Objective:**

To determine whether initiation of semaglutide is associated with fewer cognitive signs/symptoms over 12 months among adults with psychiatric disorders.

**Methods:**

Retrospective cohort study using de-identified multicentre US electronic health records (EHRs) in NeuroBlu Data spanning 1999–2024. We included adults with any International Classification of Diseases (ICD)-9/-10 psychiatric diagnosis with at least one semistructured, clinician-rated routine cognitive assessment in the 12 months before and at least one in the 12 months after initiating an antidiabetic drug. Semaglutide initiation was compared against four comparator strategies including sitagliptin, empagliflozin, glipizide or no antidiabetic treatment. Primary outcome was a 0–100 composite cognitive score (0, no signs/symptoms recorded; 100, signs/symptoms present across all domains) derived from semistructured, clinician-rated routine cognitive assessments across memory, attention, orientation and other cognitive functions, with a 12-month follow-up after antidiabetic drug initiation. We estimated mean ratios (MR) and 95% CIs between comparisons with the parametric g-formula, adjusting for baseline cognitive score and prespecified covariates. Additional analyses were conducted for individual cognitive domains and also grouped by major depression, psychosis and bipolar disorder.

**Findings:**

Among 13 007 individuals (mean (SD) age, 62.0 (13.4) years; 6300 (48.4%) female), 1261 initiated semaglutide. Across psychiatric disorders, mean cognitive signs/symptoms scores over 12 months were lower with semaglutide (11.14) than with no treatment (14.91; MR, 0.75 (0.65–0.85); Bonferroni-corrected p=0.00002), glipizide (13.46; MR 0.83 (0.70–0.93); p=0.041) or empagliflozin (13.69; MR, 0.81 (0.69–0.94); p=0.016) and lower but not significantly different from sitagliptin (12.52; MR, 0.89 (0.77–1.02); p=0.50). Associations were stronger for memory and broader cognitive functions. Similar effect sizes were seen across disorders but were only consistently significant in major depression.

**Conclusions:**

In this EHR-based cohort of adults with psychiatric disorders, semaglutide initiation was associated with lower clinician-recorded cognitive signs/symptoms than several comparators over 12 months.

**Clinical implications:**

Randomised clinical trials are needed to confirm causality and clinical utility of semaglutide to reduce cognitive signs/symptoms in psychiatric disorders.

**WHAT IS ALREADY KNOWN ON THIS TOPIC**

**WHAT THIS STUDY ADDS**

- In this retrospective cohort study of electronic health records including 13 007 patients in the USA with any psychiatric diagnosis, 12-month cognitive signs and symptoms scores were lower with semaglutide than with empagliflozin, glipizide or no treatment but similar to sitagliptin.
- The most favourable associations for semaglutide involved memory and broader cognitive functions and were observed in people with major depressive disorder.

**HOW THIS STUDY MIGHT AFFECT RESEARCH, PRACTICE OR POLICY**

- Semaglutide use is associated with better cognitive signs and symptoms in psychiatric populations, supporting confirmatory randomised trials.

## Background

 Cognitive signs and symptoms are frequently observed in several psychiatric conditions,^1^ affecting between one- and two-thirds of people with psychosis and mood disorders.^2^ These impairments are burdensome^3–5^ and poorly addressed by existing treatments.^5 6^ More than a decade after a call for novel therapeutic approaches to target cognitive dysfunction,^7^ available interventions remain unsatisfactory.

While glucagon-like peptide-1 receptor agonists (GLP-1RAs) such as semaglutide have been developed to treat type-2 diabetes mellitus (T2DM) and obesity, GLP-1 receptors are highly present in the brain, including in systems involved in cognition.^8^ Their pro-cognitive potential has been suggested in pre-clinical models^9^ and supported by pharmacoepidemiological studies in people with T2DM.^10 11^ While two large clinical trials have been conducted in Alzheimer’s disease with mixed results,^12 13^ clinical trials assessing cognitive effects of GLP-1RAs in T2DM have been mostly small.^14^ The only larger trial reported a 14% reduction in cognitive impairment with the GLP-1RA dulaglutide after post hoc adjustment for covariates.^15^

In people with a psychiatric disorder, GLP-1RAs may thus offer a promising approach to improving cognitive outcomes, in addition to their other metabolic benefits.^16^ Few clinical trials have examined the cognitive effects of semaglutide in individuals with mental illness, and no large observational study has assessed these associations across psychiatric diagnoses using routinely collected clinical data. It cannot be assumed that findings from the general population extend to populations with a psychiatric condition since mechanisms underlying their cognitive impairment likely differ.^6^ In addition, no study has investigated the effect of semaglutide across specific cognitive domains, which is important to better understand who would most benefit from such treatments.

### Objective

In this study, we begin to fill these gaps by analysing time-varying, symptom-level data in a large electronic health records (EHRs) dataset. Specifically, we assess the cognitive signs and symptoms across different domains within the first 12 months since initiating semaglutide and compare them to cognitive signs and symptoms in people receiving other antidiabetic medications. Based on our prior research^10^ and supporting translational and clinical evidence,^8 9 11 12 15^ we hypothesised that semaglutide would be associated with better cognitive outcomes in this population.

## Methods

### Study design and data collection

For this retrospective cohort study, we used de-identified EHR data from NeuroBlu (V24R5), a longitudinal behavioural health real-world database comprising records of over 32 million patients collected during routine healthcare provision across multiple specialty mental healthcare organisations in the USA during the calendar period 1999–2024.^17^ Patients are included in NeuroBlu Data if they have at least one recorded psychiatric condition of any type; for them, all available medical records in the EHR system (not limited to psychiatric care) are collected. These records have been standardised to the Observational Medical Outcomes Partnership Common Data Model.

This observational study was not pre-registered. The exposure, comparator treatment strategies, covariates and analytical approach were selected before the analyses were conducted and were informed by prior pharmacoepidemiological work on semaglutide and cognitive outcomes.^10^

### Ethics statement

NeuroBlu Data has received a waiver of authorisation to analyse de-identified healthcare data from the Western Institutional Review Board-Copernicus Group (reference: WCG-IRB 1–1470336-1). Therefore, this study did not require further ethical approval and individual patient consent. See also online supplemental eMethods.

### Cohorts’ definition

The study population included participants who met the following inclusion criteria:

- Diagnosis of any psychiatric condition recorded in their EHR (ICD-10-CM codes F00-F99 or ICD-9-CM codes 290–319). Diagnostic subgroup analyses were then conducted for major depressive disorder, psychotic disorders and bipolar disorder.
- At least one recorded prescription of either semaglutide (exposed cohort) or a comparable antidiabetic medication (empagliflozin, glipizide or sitagliptin) as per our previous study,^10^ after or on the day of their first recorded psychiatric diagnosis. Treatment exposure was based on recorded prescriptions in the EHR, not dispensing records or confirmed medication adherence. A ‘no treatment’ strategy was estimated from individuals who discontinued antidiabetic medication during follow-up, rather than from individuals who never received antidiabetic medication (see Analysis section below).
- At least one semistructured, clinician-rated routine cognitive assessment recorded within the 12 months before initiation of an antidiabetic drug and at least another one recorded within the 12 months after initiation.

Patients with a diagnosis of dementia before antidiabetic exposure were excluded. Detailed definitions including ICD-9/10 codes are provided in online supplemental table 1.

The index date was defined as the time of the first recorded semaglutide prescription for the primary cohort and the first recorded prescription of sitagliptin, empagliflozin or glipizide, respectively, for the comparator cohorts.

### Covariates

Covariates were selected based on expert opinion and prior literature^10 11^ and included factors associated with both the choice of antidiabetic medication and cognitive outcomes, namely demographic variables (sex assigned at birth, age, self-reported race), baseline cognition score, psychiatric comorbidities (ICD-9 and ICD-10 codes for substance use disorders, psychotic disorders, mood disorders and anxiety disorders), physical comorbidities (ICD-9 and ICD-10 codes for cardiovascular disease (CVD) and metabolic disorders, including diabetes) and concomitant prescriptions of antipsychotics, antidepressants, cardiovascular drugs and other antidiabetic drugs. Details on diagnostic codes and medication classes are provided in online supplemental tables 1,2. Apart from sex assigned at birth and self-reported race, all covariates were considered to be time-varying (eg, someone might only be on a concomitant cardiovascular drug for part of the follow-up).

### Outcomes

The primary outcome was a composite cognitive score ranging from 0% to 100% and derived from semistructured, clinician-rated routine cognitive assessments as follows.

Clinicians reported their findings from routine cognitive assessments in a standardised form within prespecified fields (one per cognitive domain) on their EHR platform. Multiple findings could be reported for each domain, and findings were either selected from a list of predefined, commonly reported findings (eg, impaired serial 7s) and/or added as free text. The four specified domains were: memory (with descriptors such as ‘only able to recall 1 of 3 items’), attention (eg, ‘impaired serial 7s’), orientation (eg, ‘disoriented to place’) and other cognitive functions (eg, ‘does not follow commands’). Across these domains, 209 descriptors were available and independently reviewed by two psychiatrists (RDG and MT) to determine whether they indicated the presence of impairment (98 descriptors, such as all four examples above), their absence (98 descriptors, such as ‘able to recall 3 of 3 items at 5 mins’) or were too ambiguous to decide either way (13 descriptors). Inter-rater reliability was estimated to have a Cohen’s κ of 0.92, and disagreements were resolved by consensus before analysis. Ambiguous descriptors were excluded from the cognitive score because they could not be clinically classified as indicating either the presence or absence of cognitive signs and symptoms. The list of included descriptors can be found in online supplemental table 3.

For each domain and individual, a score of 0 was recorded if all descriptors indicated the absence of cognitive signs and symptoms, and a score of 1 was recorded if at least one descriptor indicated their presence. If no descriptor was present for a given domain, a missing value was recorded. The composite cognitive score was defined as the average of scores across cognitive domains expressed in percents, after excluding missing values for an individual, which is equivalent to imputing the missing values with the mean of the other domains at that time point for each individual. Secondary outcomes included scores for each cognitive domain independently.

### Analysis

Because the exposures and several covariates (eg, other prescribed medications) are time-varying (ie, they vary over the follow-up period), we used the parametric g-formula, a well-established approach to estimating time-varying effects, and implemented in gfoRmula R package v. 1.11^18^ to estimate the effect of antidiabetic drugs on cognitive scores.^19^ The g-formula allows estimation of the effect of different treatment strategies, defined as sequences of treatment selection over time. Using all available data, the g-formula models the data-generating process over follow-up, including how covariates evolve and how outcomes arise given past treatment and covariate history, and then uses this model to estimate the outcome expected under a specified treatment strategy. In theory, any treatment strategy can be modelled, including complex strategies involving switching or stopping treatment. In practice, we focused on *static* treatment strategies, namely strategies in which the same treatment is selected throughout follow-up.

We compared the cognitive score after the strategy ‘always treat with semaglutide’, corresponding to sustained semaglutide treatment throughout the 12-month follow-up, with four comparator strategies: ‘always treat with sitagliptin’, ‘always treat with empagliflozin’, ‘always treat with glipizide’ and ‘no treatment’ (ie, receiving no antidiabetic drug during follow-up). Observed treatment status was coded as time-varying in the data used to fit the g-formula models, so treatment discontinuation and switching contributed to the estimated relationships between prior treatment, covariates and subsequent outcomes. The static strategy ‘always treat with semaglutide’ should therefore be interpreted as a modelled counterfactual sustained-treatment strategy, not as a description of the observed mean duration of semaglutide exposure. Observed treatment was coded monthly as time-varying, so individuals contributed information during the months in which they received each treatment. The 12-month ‘always treat’ estimates combined these observed histories across follow-up and should be interpreted as modelled sustained-treatment strategies rather than observed mean treatment durations (see online supplemental eMethods). Switching between antidiabetic medications during follow-up was captured through monthly time-varying treatment indicators. No lag or washout period was applied, because dispensing, adherence, dose, formulation and residual biological exposure could not be reliably inferred from the EHR prescription records. Since being on an antidiabetic drug at baseline is an inclusion criterion, the effect of the ‘no treatment’ strategy is estimated from people who stopped taking an antidiabetic drug during follow-up. This comparison should be considered exploratory because discontinuation may reflect clinical factors that are not fully captured in the EHR.

Time-varying variables were represented as binary time series by dividing the 12-month follow-up into 12 discrete time periods of 1 month each, with presence or absence of exposure/covariates coded as 1 and 0, respectively, except for cognitive score (that was a continuous variable, and averaged over the month if multiple cognitive assessments were recorded within that month) and age (that was also considered a continuous variable). Age and exposure had no missing data. Categorical variables with missing values (eg, self-reported race) had ‘unknown’ values as a separate category since this might in part represent demographically distinct subgroups (eg, people who do not identify with a specific race) (see online supplemental file, eMethods).

Results are presented as mean ratios (MR), namely ratios of the mean cognitive score at the end of follow-up between semaglutide and comparator treatment strategies; 95% CIs were obtained using bootstrapping with 100 repetitions. P values for the hypothesis that there is no difference between semaglutide and other treatment strategies were obtained using a normal-approximation bootstrap in which the SD of the difference in mean cognitive scores is estimated from bootstrap samples to derive z-scores. This approach was preferred over simple percentile bootstrap as the latter is unstable for low P values and small number of bootstrap samples (which was constrained here by the computational cost of running the g-formula in our large sample). P values for the primary analyses, including for individual cognitive domains, were Bonferroni-corrected for the four comparisons. Two-sided P values <0.05 were considered statistically significant.

All analyses were repeated after restricting the cohort to the three most common diagnoses: major depressive disorder, psychotic disorders and bipolar disorder.

## Findings

### Cohorts’ characteristics

A total of 13 007 patients were included in the study (mean (SD) age 62.0 (13.4) years; 6300 (48.4%) females; 8380 (64.4%) White and 3657 (28.1%) Black or African American race; 5679 (43.7%) with major depressive disorder, 2251 (17.3%) with psychotic disorders, 1825 (14.0%) with bipolar disorder; see table 1 for detailed characteristics), of whom 1261 were on semaglutide, 4479 on empagliflozin, 4890 on glipizide and 3462 on sitagliptin (summing to more than the total sample size due to switching between antidiabetic drugs during follow-up or on multiple antidiabetics concurrently). Antipsychotics (7018 individuals, 54.0%), antidepressants (9496 individuals, 73.0%), cardiovascular (12 521 individuals, 96.3%) and other metabolic medications (12 600 individuals, 96.9%) were commonly co-prescribed. At baseline, 3611 (27.8%) individuals showed cognitive signs and symptoms (mean cognitive score 10.83 (22.42)).

**Table 1:** – Baseline characteristics of the whole cohort and the cohorts restricted to major depressive disorder, bipolar disorder and psychotic disorders

Characteristics	Total population	Major depressive disorder	Psychotic disorders	Bipolar disorder
*Cohort size*	13 007	5 679	2 251	1 825
*Demographics*				
Age at baseline, years	62.0 (13.4)	60.1 (13.4)	58.1 (13.7)	52.9 (11.7)
Sex				
Female	6 300 (48.4%)	3 284 (57.8%)	1 136 (50.5%)	1 074 (58.8%)
Male	6 707 (51.6%)	2 395 (42.2%)	1 115 (49.5%)	751 (41.2%)
Race				
White	8 380 (64.4%)	3 871 (68.2%)	1 273 (56.6%)	1 152 (63.1%)
Black or African American	3 657 (28.1%)	1 379 (24.3%)	738 (32.8%)	490 (26.8%)
Other race	490 (3.8%)	231 (4.1%)	104 (4.6%)	79 (4.3%)
Unknown race	480 (3.7%)	198 (3.5%)	136 (6.0%)	104 (5.7%)
*Clinical features*				
Baseline cognitive signs and symptoms	3 611 (27.8%)	1 589 (28.0%)	986 (43.8%)	704 (38.6%)
Baseline cognitive score	10.83 (22.42)	10.46 (21.41)	19.70 (28.09)	16.70 (25.94)
*Treatment*				
Semaglutide Exposure duration, months	1 261 (9.7%)	648 (11.4%)	152 (6.8%)	171 (9.4%)
	4.75 (3.87)	4.78 (3.69)	5.10 (3.95)	5.03 (3.91)
Empagliflozin Exposure duration, months	4 479 (34.4%)	1 631 (28.7%)	498 (22.1%)	471 (25.8%)
	6.04 (4.89)	6.01 (4.73)	5.27 (4.53)	5.80 (4.61)
Sitagliptin Exposure duration, months	3 462 (26.6%)	1773 (31.2%)	760 (33.8%)	587 (32.2%)
	5.59 (4.46)	5.71 (4.28)	5.41 (4.44)	5.74 (4.41)
Glipizide Exposure duration, months	4 890 (37.6%)	1 976 (34.8%)	978 (43.4%)	720 (39.5%)
	4.22 (4.09)	4.52 (4.04)	4.63 (4.14)	4.59 (4.13)
Antipsychotics Exposure duration, months	7 018 (54.0%)	3 652 (64.3%)	2 186 (97.2%)	1 675 (91.8%)
	3.60 (3.69)	3.68 (3.58)	4.97 (4.06)	4.85 (3.88)
Antidepressants Exposure duration, months	9 496 (73.0%)	5 324 (93.7%)	1 811 (80.5%)	1 640 (89.9%)
	5.14 (4.13)	5.68 (4.04)	5.05 (4.02)	5.28 (4.07)
Cardiovascular medications Exposure duration, months	12 521 (96.3%)	5 443 (95.8%)	2 123 (94.3%)	1 709 (93.6%)
	7.06 (4.22)	6.75 (4.07)	6.16 (4.17)	6.09 (4.20)
Other antidiabetics Exposure duration, months	12 600 (96.9%)	5 465 (96.2%)	2 151 (95.6%)	1 740 (95.3%)
	5.89 (4.19)	5.93 (4.07)	5.66 (4.10)	5.69 (4.12)

Data are n (%) or mean (SD). ‘Other Race’ comprises categories of Asian, American Indian/Alaska Native or Native Hawaiian/Pacific Islander; ‘Unknown Race’ refers to patients with no matching race concept. Time under exposure is reported for patients who had the exposure recorded and includes both baseline and follow-up.

### Cognitive outcomes

**Figure 1:**
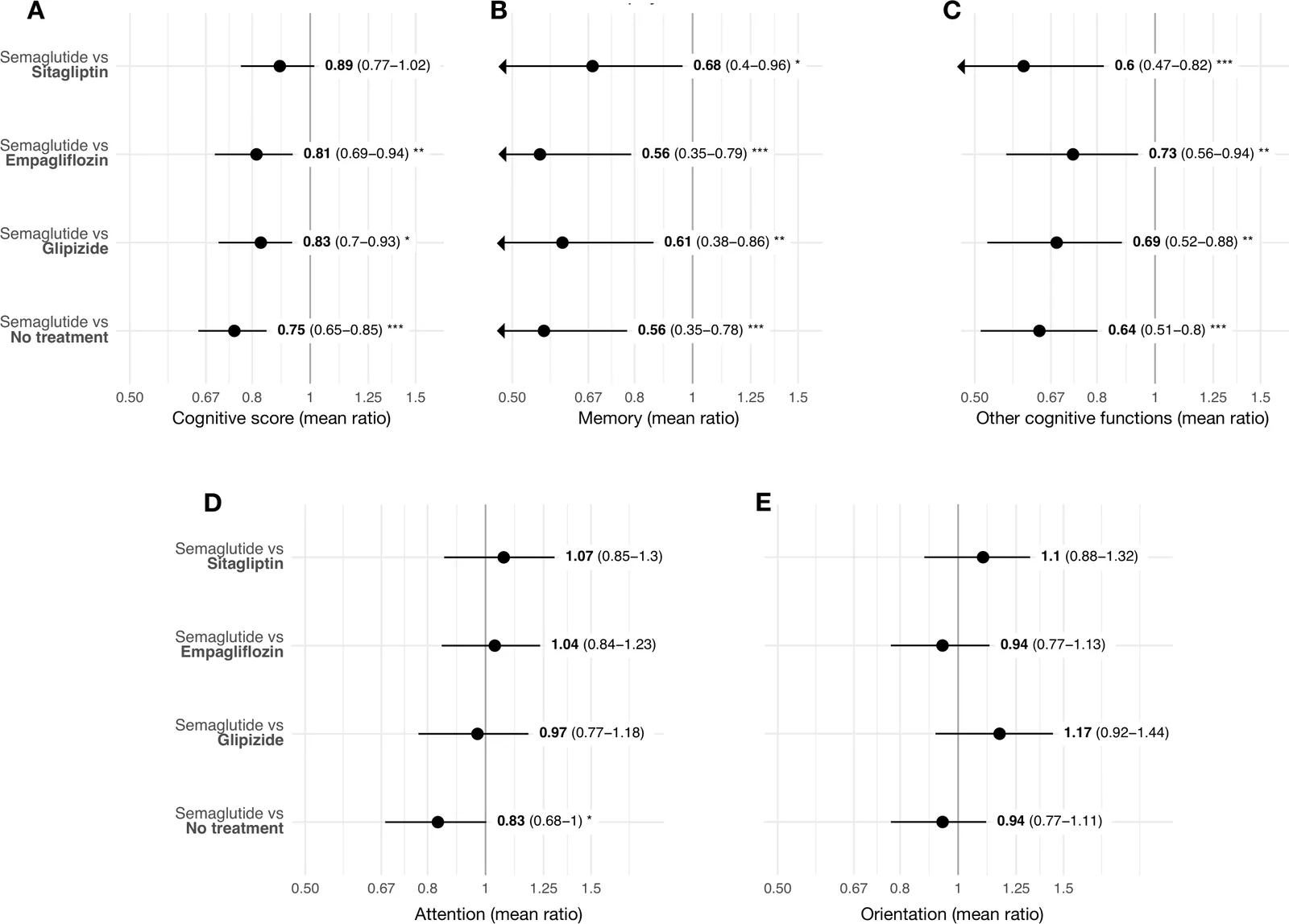
Mean ratios (MR) of cognitive signs and symptoms between semaglutide and other treatment strategies in terms of (**A**) composite cognitive score across domains, (**B**) memory, (**C**) other cognitive functions, (**D**) attention and (**E**) orientation. Legend: MR <1 indicates fewer cognitive signs and symptoms in the semaglutide treatment strategy. The dots and numbers in bold indicate the point estimate of the MR, whereas horizontal lines and numbers in brackets indicate the CI. *p<0.05, **p<0.01, ***p<0.001; all adjusted for multiple comparisons.

As shown in figure 1A and table 2, semaglutide was associated with significantly better cognitive signs and symptoms than no treatment (MR, 0.75; 95% CI 0.65 to 0.85, mean cognitive score at 12 months of 14.9 in those with no treatment versus 11.1 in those on semaglutide, p<0.0001 after Bonferroni-correction for multiple comparisons), glipizide (MR, 0.83; 95% CI 0.70 to 0.93; mean score of 13.5 vs 11.1, Bonferroni-corrected p=0.041) and empagliflozin (MR, 0.81; 95% CI 0.69 to 0.94; mean score of 13.7 vs 11.1, Bonferroni-corrected p=0.016) but similar cognitive scores as sitagliptin (MR, 0.89; 95% CI 0.77 to 1.02; mean score of 12.5 vs 11.1, Bonferroni-corrected p=0.50).

**Table 2:** – Results of the analysis in the whole cohort for the comparison between semaglutide and the other four comparator strategies (no treatment and always treat with glipizide, empagliflozin and sitagliptin)

Outcome	Comparator	Mean ratio (95% CI)	Mean score in comparator cohort	Mean score in semaglutide cohort	p Value	p Value (adjusted)
***Cognitive score***	No treatment	0.75 (0.65 to 0.85)	14.91	11.14	4.1×10^–6^	0.00002
	Glipizide	0.83 (0.70 to 0.93)	13.46	11.14	0.01	0.041
	Empagliflozin	0.81 (0.69 to 0.94)	13.69	11.14	0.004	0.016
	Sitagliptin	0.89 (0.77 to 1.02)	12.52	11.10	0.12	0.50
*Memory*	No treatment	0.56 (0.35 to 0.78)	37.92	21.42	0.0001	0.001
	Glipizide	0.61 (0.38 to 0.86)	35.32	21.42	0.002	0.01
	Empagliflozin	0.56 (0.35 to 0.79)	38.50	21.42	0.0003	0.001
	Sitagliptin	0.68 (0.40 to 0.96)	31.47	21.42	0.03	0.11
*Other cognitive functions*	No treatment	0.64 (0.51 to 0.80)	16.17	10.37	1.3×10^–5^	0.0001
	Glipizide	0.69 (0.52 to 0.88)	15.14	10.37	0.002	0.01
	Empagliflozin	0.73 (0.56 to 0.94)	14.21	10.37	0.01	0.04
	Sitagliptin	0.60 (0.47 to 0.82)	17.18	10.37	0.0002	0.001
*Attention*	No treatment	0.83 (0.68 to 1.00)	17.71	14.75	0.05	0.18
	Glipizide	0.97 (0.77 to 1.18)	15.21	14.75	0.77	1.00
	Empagliflozin	1.04 (0.84 to 1.23)	14.23	14.75	0.73	1.00
	Sitagliptin	1.07 (0.85 to 1.30)	13.75	14.75	0.54	1.00
*Orientation*	No treatment	0.94 (0.77 to 1.11)	9.56	9.01	0.56	1.00
	Glipizide	1.17 (0.92 to 1.44)	7.68	9.01	0.20	0.79
	Empagliflozin	0.94 (0.77 to 1.13)	9.56	9.01	0.56	1.00
	Sitagliptin	1.10 (0.88 to 1.32)	8.18	9.01	0.39	1.00

Mean ratio represents the ratio of mean cognitive scores at 12 months in the semaglutide cohort over that in the comparator treatment strategy. The mean scores in both groups are reported as are the unadjusted and Bonferroni-adjusted p-values. All results across diagnostic subgroups are presented in online supplemental table 4.

Similar findings were observed in terms of memory (significantly better after semaglutide than all other treatment strategies: MRs between 0.56 and 0.61, all Bonferroni-corrected p<0.01 except for the comparison with sitagliptin: MR, 0.68; 95% CI 0.40 to 0.96; p=0.11; figure 1B) and other cognitive functions (significantly better after semaglutide than all other treatment strategies: MRs between 0.60 and 0.73, all Bonferroni-corrected p<0.05; figure 1C). No significant difference in attention and orientation was observed between semaglutide and other treatment strategies (figure 1D,E). All results including MRs and mean cognitive scores for each domain and comparison are presented in figure 1 and table 2.

**Figure 2:**
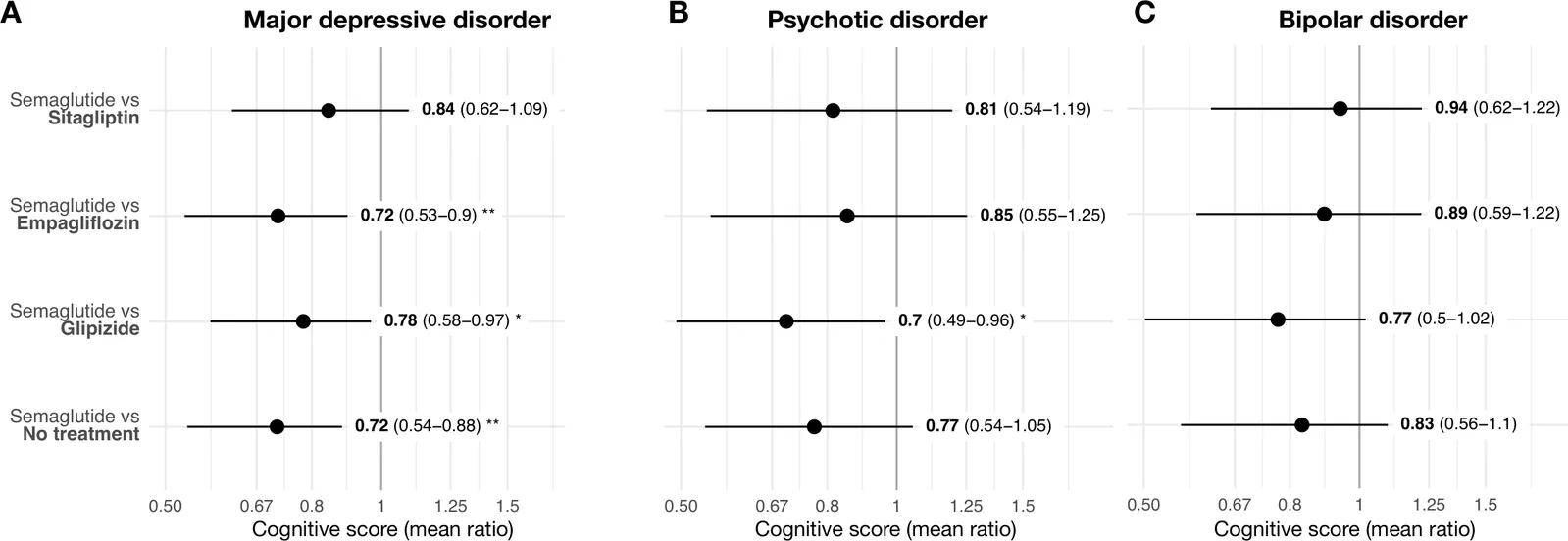
Mean ratios (MR) of cognitive signs and symptoms between semaglutide and other treatment strategies in terms of composite cognitive score among people with a diagnosis of (**A**) major depressive disorder, (**B**) psychotic disorders and (**C**) bipolar disorder. Legend: MR <1 indicates fewer cognitive signs and symptoms in the semaglutide treatment strategy. The dots and numbers in bold indicate the point estimate of the MR, whereas horizontal lines and numbers in brackets indicate the CI. *p<0.05, **p<0.01, ***p<0.001.

Comparable associations were observed when the cohort was restricted to people with major depressive disorder, with significantly better cognitive scores after semaglutide than after all other treatments (MRs between 0.72 and 0.78, all p<0.05), except sitagliptin (figure 2A). Similar effect sizes were observed in psychotic and bipolar disorders, but most did not reach statistical significance (figure 2B,C). Outcomes for all cognitive domains and all diagnostic subgroups are shown in online supplemental table 4.

## Discussion

This retrospective cohort study using de-identified EHRs provides the first large-scale real-world clinical evidence in a psychiatric population that semaglutide is associated with fewer cognitive signs and symptoms within 1 year of treatment initiation.

The absolute difference in cognitive signs and symptoms is clinically meaningful. At 12 months, the average cognitive score was 14.9 in those not receiving antidiabetic treatment, approximately equivalent to the score that would be observed if 60 out of 100 people showed signs and symptoms in one cognitive domain (see online supplemental file, eMethods). In the semaglutide group, the average score was 11.1, which would be observed if 15 of these 60 people no longer showed any cognitive signs and symptoms. The difference is even greater in specific cognitive domains (figure 1B,C, table 2). For example, around 35 out of 100 people had memory signs and symptoms in the empagliflozin, glipizide or untreated groups, compared with just 21 out of 100 on semaglutide.

Positive findings were more robustly observed in terms of memory and other cognitive functions, while little to no effect was observed for attention and orientation. The relatively narrow CIs around null effects, especially for orientation, suggest that these represent true negative findings rather than lack of statistical power. These differential associations might reflect differing mechanisms underpinning signs and symptoms in these domains; for instance, disorientation and lack of attention are common features of acute confusional state, whereas impairment in memory and other cognitive functions might reflect more sustained cognitive deficits. Our findings can therefore help design confirmatory clinical trials by targeting individuals with deficits in domains most likely to improve with GLP-1RAs.

The clearest diagnostic subgroup findings were observed in major depressive disorder, and corroborate those from a recent randomised controlled trial in 72 people with depression that reported treatment effects of semaglutide on global cognition.^20^ In people with psychotic and bipolar disorders, the point estimates of cognitive outcomes remained similar to those seen in the whole cohort, but the comparisons rarely reached statistical significance. This may reflect a lack of statistical power as these subgroups were smaller. It is also possible that the effect is restricted to major depressive disorder due to differing cognitive profiles and different mechanisms underpinning them. Indeed, attention is known to be particularly impaired in bipolar disorder,^21^ but was not observed to be consistently affected by semaglutide in this study. If the effect was restricted to major depressive disorder, it might still represent significant progress in the management of cognitive impairment in psychiatry, given that depression is a common comorbidity of many other psychiatric conditions and might therefore contribute to the overall cognitive burden across diagnoses. If confirmed in adequately powered clinical trials with objective cognitive testing, these findings would provide complementary real-world evidence that strengthens the case for repurposing GLP-1RAs to manage cognitive deficits across psychiatric conditions.

While semaglutide was associated with significantly fewer cognitive signs and symptoms than most comparators, it showed similar outcomes to the DPP-4 inhibitor sitagliptin, except for more favourable outcomes in the ‘other cognitive functions’ domain. One possibility is that both semaglutide and sitagliptin preserve cognition through partly shared metabolic or glycaemic optimisation, rather than through a semaglutide-specific neuroprotective effect. This contrasts with findings in the general population with T2DM, where semaglutide was linked to a lower risk of cognitive deficits compared with sitagliptin.^10^ Such apparent discrepancy may reflect differences in population structure and highlights the importance of assessing effects in psychiatric populations. It may also relate to differences in outcome definitions: the general population study captured clinical diagnoses (e.g. mild cognitive impairment),^10^ which likely reflect more severe impairment, while the present study focused on cognitive status, potentially identifying milder but more pervasive deficits common in psychiatric conditions. Supporting this interpretation, a Danish study found that GLP-1RAs reduced the risk of major but not minor cognitive impairment compared with DPP-4 inhibitors.^22^ Pharmacologically, DPP-4 inhibitors inhibit the enzyme that degrades endogenous GLP-1, whereas GLP-1RAs more directly and effectively activate GLP-1 receptors.^23^ Taken together, these findings support the role of the GLP-1 system in cognitive functioning^8^ and suggest that GLP-1RAs may offer more robust benefits than DPP-4 inhibitors for more pronounced cognitive impairment. However, we could not assess HbA1c, weight change, insulin resistance or inflammatory markers with sufficient completeness in NeuroBlu, and therefore cannot distinguish direct GLP-1 receptor-mediated effects from effects mediated by improved metabolic health. Further research is needed to clarify the underlying mechanisms.

One exploratory finding moved in the opposite direction: among people with bipolar disorder, semaglutide was associated with higher signs and symptoms in the attention domain than empagliflozin. This was not observed for the composite score or for comparisons with the other treatment strategies, and it arose in a secondary subgroup-domain analysis. It should therefore be interpreted cautiously, but it argues against assuming uniform cognitive benefit across all domains and diagnostic groups.

### Limitations

This study has several strengths including a large sample size, the use of the g-formula to account for time-varying confounding factors, the investigation of cognitive signs and symptoms across and within major psychiatric diagnoses, and the assessment of cognition across different domains. However, it also has limitations.

First, this is an observational study and remains prone to unmeasured confounding, channelling and ascertainment bias. NeuroBlu does not systematically capture socioeconomic status, insurance status, lifestyle factors or all markers of healthcare access, and differences in clinical contact may affect the likelihood that cognitive signs and symptoms were recorded. The requirement for cognitive assessments before and after treatment initiation may also have selected individuals with greater clinical contact, cognitive concerns or psychiatric severity, thereby limiting generalisability, although the same inclusion criterion was applied across treatment groups. Psychiatric illness severity may also influence both treatment choice and cognitive outcomes; although we adjusted for psychiatric comorbidities, baseline cognitive score and concomitant psychotropic prescriptions, EHR data do not consistently capture illness duration, symptom burden, functioning or treatment resistance. Cognitive assessments were less frequent during semaglutide exposure than during exposure to the comparator drugs (see online supplemental file, eMethods), arguing against more frequent ascertainment as an explanation for the observed association. However, this does not exclude bias related to differential assessment timing or recording practices. Lower assessment frequency therefore required greater reliance on interpolation and carry-forward/backward imputation, which may attenuate temporal changes, although the direction of any resulting bias cannot be established with certainty. We also adjusted for concomitant antipsychotic and antidepressant prescriptions, but did not calculate a formal anticholinergic load. The absence of a comparable association with orientation provides some reassurance against a wholly non-specific recording artefact, but does not exclude domain-specific unmeasured confounding.

Second, cognition was assessed using semi-structured, clinician-rated routine cognitive assessments, which lack the granularity and objectivity of standardised cognitive testing. This imprecision is more likely to increase variance and bias estimates towards the null, unless recording differed systematically between treatment groups. The composite cognitive score was clinically derived and has not been formally validated against standardised neuropsychological instruments. Notably, the burden of cognitive signs and symptoms as measured by the composite score at baseline was highest in psychotic disorders, as expected. Although descriptor classification showed high inter-rater reliability and had face and content validity, future studies should assess its concurrent validity against established cognitive measures.

Third, treatment exposures were inferred from EHR prescription records. We could not assess dispensing, adherence, dose, formulation or residual biological exposure after switching. Likewise, we could not reliably distinguish oral from subcutaneous semaglutide. These formulations differ in bioavailability, dosing and clinical use, although both contain the same active molecule and act through the same GLP-1 receptor pathway; pooling would be expected to dilute formulation-specific effects rather than create a spurious protective association. The findings should therefore be interpreted as reflecting real-world semaglutide prescribing overall rather than either formulation specifically.

Fourth, differences in antidiabetic treatment costs and market availability might have influenced treatment selection, although our supplementary analyses suggest that these factors are unlikely to explain the findings. Fifth, the g-formula can estimate outcomes under treatment strategies that are not fully observed in individuals. This is relevant to the ‘no treatment’ strategy, which was estimated from individuals who discontinued antidiabetic medication during follow-up, and to static sustained-treatment strategies, including ‘always treat with semaglutide’, especially because the mean recorded duration of semaglutide exposure was shorter than 12 months. These estimates depend partly on model-based extrapolation and should be interpreted cautiously, particularly for the magnitude of 12-month sustained-treatment effects. Sixth, results are limited to people prescribed antidiabetic medication in routine care. Whether similar cognitive associations would be observed in younger people, people without diabetes or those receiving GLP-1RAs primarily for weight management requires prospective study. Seventh, weight loss and improved metabolic health may mediate the association between semaglutide and lower cognitive signs and symptoms. Assessing the contribution of these mediating pathways in the observed association between semaglutide and cognitive function will require future work as it involves sophisticated statistical analyses to account for time-varying confounders in the mediation analysis. Finally, our findings are limited to semaglutide. Although the literature suggests a potential class effect on cognition, extrapolation to other GLP-1RAs requires further supporting evidence.^24^

### Clinical implications

We provide evidence that semaglutide is associated with lower burden of cognitive signs and symptoms in people with a psychiatric condition. These findings are relevant in the context of emerging clinical guidelines on the inclusion of GLP-1RAs within treatment algorithms for obesity in individuals with severe mental illness. However, direct extrapolation to this broader clinical population requires caution. The cohort in this study was older, with a mean age of 62 years, and included individuals prescribed antidiabetic medication in routine care. Whether similar cognitive associations would be observed in younger people, in those without diabetes, or in those receiving GLP-1RAs primarily for weight management remains unknown. If confirmed in clinical trials, semaglutide could be used as a novel pharmacological intervention to reduce the cognitive burden of several psychiatric disorders, positively impacting individual patients, healthcare providers and the broader society.

## Supplementary material

10.1136/bmjment-2026-302828 online supplemental file 1

## Data Availability

Data may be obtained from a third party and are not publicly available.
