# Supplementary material for: Cognitive signs and symptoms in people with a psychiatric diagnosis on semaglutide: a retrospective cohort study of 13 007 patients in the USA
Source: BMJ Ment Health. 2026 Aug 20;29(1):e302828. doi: 10.1136/bmjment-2026-302828 (PMC13504872; doi:10.1136/bmjment-2026-302828)
Supplement: Supplementary file 1 [file bmjment-29-1-s001.docx]

**ONLINE SUPPLEMENTAL FILE**

This online supplement file forms part of the original submission and is peer-reviewed. Supplement to: De Giorgi R, Lipunova N, Mathias E, Shang F, Taquet M. **Cognitive signs and symptoms in people with a psychiatric diagnosis on semaglutide: a retrospective cohort study of 13,007 patients in the United States**

[Online supplemental file, eMethods	2](#_Toc234592751)

[Data source description	2](#_Toc234592752)

[Demographic profile	2](#_Toc234592753)

[Clinical setting	4](#_Toc234592754)

[Recording of non-psychiatric care	4](#_Toc234592755)

[Data management	5](#_Toc234592756)

[Missing data	5](#_Toc234592757)

[Interpretation of effect sizes	6](#_Toc234592758)

[Dealing with selection and time period bias	6](#_Toc234592759)

[Dealing with ascertainment and channelling bias	7](#_Toc234592760)

[Support for sustained treatment strategies	8](#_Toc234592761)

[Online supplemental file, table S1. Operational definitions for disorders and disorder groups	9](#_Toc234592762)

[Online supplemental file, table S2. Medication classes and individual compounds included in each class	10](#_Toc234592763)

[Online supplemental file, table S3. Operational definition of cognitive status using routinely collected cognitive assessment records in NeuroBlu Data V24R5	11](#_Toc234592764)

[Online supplemental file, table S4. Results for all cognitive domains and for all cohorts including the whole cohort (referred to as ‘All psychiatric disorders’)	16](#_Toc234592765)

# Online supplemental file, eMethods

## Data source description

The study population was selected from Holmusk’s NeuroBlu Data (V24R5), a longitudinal behavioural health real-world database comprising structured and unstructured patient-level clinical data derived from de-identified electronic health records (EHRs) primarily from specialty behavioural health care settings in the United States of America (USA). The data in the NeuroBlu Data have been collected during provision of routine healthcare across multiple institutions in the USA throughout years 1999-2024. Patients were included in the NeuroBlu Data if they were recorded with at least one mental health diagnosis of any type.

## Demographic profile

In the total population of NeuroBlu Data (N=32,936,960, V24R5), there are slightly more females than males (58% females). Where patient race is known (79%), most patients are recorded to be White (58%), followed by Black or African American (9.8%), with small proportions of patients of other race groups. NeuroBlu Data contain patients of all ages with the highest proportion of patients being 21-60 years old at the time of their first recorded visit. NeuroBlu Data contain patients from 49 USA states (i.e., all but Hawaii) and Washington DC, with a breakdown provided below:

| **State** | **Proportion of patients in the NeuroBlu data** |
| --- | --- |
| AK | 0.22% |
| AL | 1.39% |
| AR | 0.68% |
| AZ | 2.66% |
| CA | 10.1% |
| CO | 2.40% |
| CT | 0.87% |
| DC | 0.10% |
| DE | 0.38% |
| FL | 7.08% |
| GA | 3.21% |
| IA | 1.29% |
| ID | 0.19% |
| IL | 2.58% |
| IN | 2.34% |
| KS | 0.49% |
| KY | 2.61% |
| LA | 0.80% |
| MA | 0.98% |
| MD | 1.86% |
| ME | 0.64% |
| MI | 3.80% |
| MN | 1.18% |
| MO | 2.08% |
| MS | 0.65% |
| MT | 0.15% |
| NC | 4.19% |
| ND | 0.14% |
| NE | 0.75% |
| NH | 0.42% |
| NJ | 1.67% |
| NM | 0.47% |
| NV | 0.68% |
| NY | 4.43% |
| OH | 6.95% |
| OK | 0.62% |
| OR | 1.14% |
| PA | 3.05% |
| RI | 0.35% |
| SC | 1.30% |
| SD | 0.09% |
| TN | 4.05% |
| TX | 7.44% |
| UT | 1.16% |
| VA | 3.35% |
| VT | 0.21% |
| WA | 1.39% |
| WI | 0.29% |
| WV | 0.53% |
| WY | 0.05% |

## Clinical setting

The database draws from a variety of care settings, including academic medical centres, community mental health clinics, non-profit health systems, private practices, and specialized substance use treatment centres. This diversity in care settings allows for a comprehensive view of mental health treatment across different modalities and patient populations.

The observation period available in NeuroBlu Data V24R5 is 1999-2024. The follow-up across patients varies significantly but is observed to be long term (i.e., 5 years and over) for 35.5% of patients (11,687,865). During the available follow-up, NeuroBlu Data captures visits that take place in emergency, inpatient, outpatient, or other (e.g., telehealth and case management visits) settings. There are no limitations to which psychiatric diagnoses are captured in NeuroBlu Data. The most common recorded diagnoses are shown to be substance-related use disorders, anxiety disorders, major depressive disorder, and bipolar disorder.

The records captured in NeuroBlu Data include diagnoses, procedures, prescriptions, healthcare provider visits, structured clinical assessments and scales, and unstructured clinical notes. The clinical notes are processed using Holmusk's proprietary natural language processing (NLP) models, extracting additional selected clinical items (e.g., anhedonia), that are otherwise not recorded in a structured format or not recorded consistently.

## Recording of non-psychiatric care

NeuroBlu Data are mostly recorded in specialized mental healthcare facilities. However, some centres are part of large health systems that provide care across a broad range of conditions. As such, the recording of non-psychiatry related prescriptions and diagnoses varies by source. In cases where the treatment of a psychiatric condition had to be supplemented with managing an organic disorder (e.g., in the setting of an emergency care), then all records for a particular patient will be retained in the dataset. In addition, if the data partner is a large healthcare system and a patient is treated across multiple departments in addition to a psychiatry-related cause, then the full set of records will be available. As the primary goal for the dataset is to capture clinical journeys of patients with mental health disorders, prescription and diagnostic data relating to organic disorders may be underreported.

## Data management

Data from healthcare providers are de-identified before being processed for quality control assessments, standardization, and analysis. All records are de-identified at source, with protected health information (PHI) removed to ensure Health Insurance Portability and Accountability Act (HIPAA) compliance and patient privacy. Institutional review board approval for studies using NeuroBlu Data is not required as the EHR data are de-identified and, thus, exempt according to the HIPAA requirements. The NeuroBlu Data has received a waiver of authorization for analysis of de-identified healthcare data from the Western-Copernicus Group (WCG) Institutional Review Board (Ref: WCG-IRB 1-1470336-1). The quality control procedures ensure that erroneous records are corrected or removed by assessing record consistency, validity, and conformance. The transformations related to quality control and standardization are guided and overseen by clinical and domain knowledge experts to ensure data reliability. All qualified records are standardized into the Observational Medical Outcomes Partnership (OMOP) Common Data Model (CDM).

## Missing data

In this study, missing values for the outcome were imputed by linear interpolation via the zoo package “na.approx” function. The function calculates the missing value by assuming a straight line between the nearest non-missing values before and after the missing value. Interpolation is only applied to internal missing values (i.e., those surrounded by non-missing values). The missing values not imputed by linear interpolation were imputed with last observation carried forward and nearest observation carried backward using the zoo package “na.locf” function.

## Interpretation of effect sizes

In the discussion, we expressed effect sizes in terms of number of people and number of domains affected. Here we describe in detail how this calculation was worked out. The outcome for a given treatment strategy is expressed as a score ranging from 0 to 100 and represents the fraction (in %) of cognitive domains affected in a group treated with such treatment strategy. In the unlikely scenario that everyone in the group show signs and symptoms in all cognitive domains, the score would be 100%. If one in five people (20%) in the group shows signs and symptoms in two of the four domains (50% of domains), the score would be 10% (= 20% × 50%). A score of 14.9 is therefore approximately equivalent to that observed if 60 out of 100 people had signs and symptoms in 1 out of 4 cognitive domains (25%) since 60 × 25% = 15. By contrast, the score of 11.1 observed under the semaglutide treatment strategy is approximately equivalent to that observed if 45 out of 100 people were still having signs and symptoms in one cognitive domain (25%) while the other 15 were no longer showing any cognitive signs and symptoms since 45 × 25% = 11.25%. This is provided for illustration of the effect size only. In our cohort we had people with signs and symptoms in 0, 1, 2, 3, and all 4 domains, and the scores of 14.9 and 11.1 result from the weighted sum of the cognitive scores across individuals.

## Dealing with selection and time period bias

Selection bias by insurance and medication cost could occur if differences in coverage and/or medication costs influenced the antidiabetic medication prescribed to people prone to cognitive signs and symptoms. There are two reasons why this potential bias is unlikely to explain our findings. First, semaglutide, like the other antidiabetic drugs, is covered by Medicaid/Medicare and is also covered by most insurance companies. Second, while the cost of semaglutide is greater than that of glipizide and sitagliptin, it is lower than that of empagliflozin according to GoodRx.com. The fact that semaglutide shows protective associations with cognitive signs and symptoms when compared to both more expensive and cheaper medications suggests that medication costs did not influence our results.

Likewise, time period bias could occur if the period during which people received their antidiabetic medication correlated with cognitive signs and symptoms. This would be possible, for instance, if there were secular trends in how cognitive signs and symptoms are recorded in mental health care, with fewer cognitive signs and symptoms being recorded recently compared to earlier periods. There is evidence against this bias in our study for two reasons. First, glipizide entered the market in 1984, sitagliptin in 2006, and empagliflozin in 2014. If secular trends accounted for our findings, we should observe that the effect of semaglutide is more marked when compared to glipizide and least marked when compared to empagliflozin, with sitagliptin having an effect that is somewhere in the middle. This does not reflect our findings, since the associations were broadly similar across comparisons with a lower effect when semaglutide was compared to sitagliptin. Second, while the dataset spans 1999-2024, most individuals in our cohort had data recorded only after 2010, with about half the patients having data recorded only after 2017, during which all treatments were available – see histogram below.

## Dealing with ascertainment and channelling bias

For each antidiabetic drug, we calculated the percentage of months during which a patient was taking that drug and in which at least one cognitive assessment was recorded. This was restricted to the 12-month follow-up period and included every patient who received the drug for at least one month within that window. We then averaged this percentage across all patients exposed to each drug. We used cogscore_raw rather than cogscore, because cogscore imputes missing monthly values by interpolation or carry-forward/backward, whereas cogscore_raw retains a true missing value when no assessment was recorded.

| **Antidiabetic drug** | **Frequency of cognitive assessments** |
| --- | --- |
| Empagliflozin | Every 2.12 months |
| Glipizide | Every 1.68 months |
| Semaglutide | Every 3.25 months |
| Sitagliptin | Every 2.05 months |

## Support for sustained treatment strategies

To assess the observed support for the sustained-treatment strategies estimated by the g-formula, we calculated the number of individuals receiving semaglutide, another antidiabetic drug, or no antidiabetic treatment in each month of the 12-month follow-up. Treatment status was time-varying, and individuals could switch between categories across months. Semaglutide exposure was observed throughout follow-up, with more than 300 individuals receiving semaglutide in every month. However, these monthly counts do not indicate that the same individuals remained continuously exposed throughout the full 12 months.

| **Follow-up month** | **Semaglutide** | **Other antidiabetic treatment** | **No antidiabetic treatment** | **Total** |
| --- | --- | --- | --- | --- |
| 1 | 1070 | 11937 | 0 | 13007 |
| 2 | 684 | 6771 | 5552 | 13007 |
| 3 | 607 | 5606 | 6794 | 13007 |
| 4 | 569 | 5441 | 6997 | 13007 |
| 5 | 478 | 4819 | 7710 | 13007 |
| 6 | 435 | 4548 | 8024 | 13007 |
| 7 | 389 | 4434 | 8184 | 13007 |
| 8 | 367 | 4348 | 8292 | 13007 |
| 9 | 355 | 4273 | 8379 | 13007 |
| 10 | 335 | 4220 | 8452 | 13007 |
| 11 | 345 | 4120 | 8542 | 13007 |
| 12 | 354 | 4069 | 8584 | 13007 |

# Online supplemental file, table S1. Operational definitions for disorders and disorder groups

| **Disorder or disorder group** | **ICD Code** | **ICD Version** |
| --- | --- | --- |
| ***Any psychiatric diagnosis*** | F00-F99. | ICD-10-CM |
|  | 290-319. | ICD-9-CM |
| ***Dementia*** | F00-F04. | ICD-10-CM |
|  | 290, 294.1, 331.19, 331.82. | ICD-9-CM |
| ***Alzheimer's disease*** | G30. | ICD-10-CM |
|  | 331.0. | ICD-9-CM |
| ***Bipolar disorder*** | F31. | ICD-10-CM |
|  | 296.0, 296.4-296.8. | ICD-9-CM |
| ***Major depressive disorder*** | F32-F33 | ICD-10-CM |
|  | 296.2-296.3. | ICD-9-CM |
| ***Substance use disorders*** | F10-F19. | ICD-10-CM |
|  | 291, 292.89, 292.9, 303-5. | ICD-9-CM |
| ***Anxiety disorders*** | F40-F41. | ICD-10-CM |
|  | 300.0, 300.2. | ICD-9-CM |
| ***Mood disorders*** | 296, 301.13, 300.4. | ICD-9-CM |
|  | F30-F34, F38-F39. | ICD-10-CM |
| ***Psychotic disorders*** | F20-F25, F28-F29, F60.1. | ICD-10-CM |
|  | 295, 297-298, 301.2. | ICD-9-CM |
| ***Cardiovascular disorders*** | I00-I99. | ICD-10-CM |
|  | 390-459. | ICD-9-CM |
| ***Metabolic disorder and diabetes*** | E08-E11, E13, E88.9. | ICD-10-CM |
|  | 249-250. | ICD-9-CM |
| ICD- International Classification of Diseases; ICD-9-CM - International Classification of Diseases, 9th Edition Clinical Modification; ICD-10-CM - International Classification of Diseases, 10th Edition Clinical Modification. | | |

# Online supplemental file, table S2. Medication classes and individual compounds included in each class

| **Medication class** | **Medications** |
| --- | --- |
| ***Antidepressants*** | Amitriptyline, Amoxapine, Bupropion, Butriptyline, Citalopram, Clomipramine, Desipramine, Desvenlafaxine, Dothiepin, Doxepin, Duloxetine, Escitalopram, Esketamine, Fluoxetine, Fluvoxamine, Imipramine, Isocarboxazid, Ketamine, Levomilnacipran, Maprotiline, Melitracen, Mianserin, Milnacipran, Mirtazapine, Nefazodone, Nortriptyline, Paroxetine, Phenelzine, Protriptyline, Reboxetine, Sertraline, Tranylcypromine, Trazodone, Trimipramine, Tryptophan, Venlafaxine, Vilazodone, Vortioxetine. |
| ***Antipsychotics*** | First-generation antipsychotics: Acepromazine, Acetophenazine, Benperidol, Bromperidol, Butaperazine, Carfenazine, Chlorpromazine, Chlorpromethazine, Chlorprothixene, Clopenthixol, Cyamemazine, Dixyrazine, Droperidol, Fluanisone, Flupentixol, Fluphenazine, Fluspirilene, Haloperidol, Lenperone, Levomepromazine, Loxapine, Mesoridazine, Molindone, Moperone, Oxypertine, Oxyprothepine, Penfluridol, Perazine, Periciazine, Perphenazine, Pimozide, Pipamperone, Piperacetazine, Pipotiazine, Prochlorperazine, Promazine, Prothipendyl, Spiperone, Sulforidazine, Thiopropazate, Thioproperazine, Thioridazine, Thiothixene, Timiperone, Tiotixene, Trifluoperazine, Trifluperidol, Triflupromazine, Zuclopenthixol. |
|  | Second-generation antipsychotics: Amisulpride, Aripiprazole, Asenapine, Blonanserin, Brexpiprazole, Cariprazine, Carpipramine, Clocapramine, Clorotepine, Clotiapine, Clozapine, Iloperidone, Levosulpiride, Lumateperone, Lurasidone, Melperone, Mosapramine, Nemonapride, Olanzapine, Olanzapine and Samidorphan, Paliperidone, Perospirone, Quetiapine, Remoxipride, Risperidone, Sertindole, Sulpiride, Sultopride, Tiapride, Veralipride, Ziprasidone, Zotepine. |
| ***Cardiovascular drugs*** | ACE inhibitors: Captopril, Cilazapril, Enalapril, Fosinopril, Imidapril, Lisinopril, Moexipril, Perindopril, Quinapril, Ramipril, Trandolapril, Benazepril. |
|  | ARBS: Azilsartan, Candesartan, Irbesartan, Losartan, Olmesartan Telmisartan, Valsartan. |
|  | Beta blockers: Acebutolol, Atenolol, Betaxolol, Bisoprolol, Esmolol, Metoprolol, Nebivolol, Carvedilol, Labetalol, Nadolol, Penbutolol, Pindolol, Propranolol, Sotalol, Timolol. |
|  | Calcium channel blockers: Amlodipine, Diltiazem, Felodipine, Isradipine, Nicardipine Nifedipine, Nisoldipine, Verapamil. |
|  | Diuretics: Chlorothiazide, Chlorthalidone, Hydrochlorothiazide, Indapamide, Metolazone, Bumetanide, Ethacrynic acid, Furosemide, Torsemide, Amiloride, Eplerenone, Spironolactone, Triamterene, Hydroflumethiazide, Ethacrynate. |
|  | Antiplatelets: Clopidogrel, Ticagrelor, Prasugrel, Cilostazol, Dipyridamole Tirofiban, Eptifibatide, Cangrelor. |
|  | Vasodilators (nitrates/alpha-2 blockers): Isosorbide Dinitrate, Terazosin, Prazosin, Doxazosin, Alfuzosin, Hydralazine. |
| ***Other antidiabetic drugs*** | Insulin, Metformin, Phenformin, Buformin, Rosiglitazone,  Pioglitazone, Troglitazone, Tolimidone, Tolbutamide, Acetohexamide, Tolazamide, Chlorpropamide, Glyburide, Glibenclamide, Glimepiride, Gliclazide, Glyclopyramide, Gliquidone, Repaglinide, Nateglinide, Miglitol, Acarbose, Voglibose, Exenatide, Eiraglutide, Albiglutide, Dulaglutide, Lixisenatide, Tirzepatide, Vildagliptin, Saxagliptin, Linagliptin, Alogliptin, Septagliptin, Teneligliptin, Gemigliptin, Pramlintide, Dapagliflozin, Canagliflozin, Remogliflozin.  *Note*: Semaglutide, empagliflozin, glipizide, and sitagliptin are excluded from this list due to them being considered as exposure/comparators in the current study. |

# Online supplemental file, table S3. Operational definition of cognitive status using routinely collected cognitive assessment records in NeuroBlu Data V24R5

| **Cognitive domain** | **Domain descriptor** | **Presence of cognitive signs and symptoms** |
| --- | --- | --- |
| ***Attention*** | Adequate | No |
|  | Appears intact | No |
|  | Appropriate attention/concentration | No |
|  | Appropriate for age attention/concentration | No |
|  | Appropriate for developmental age | No |
|  | Decreased attention/concentration | Yes |
|  | Difficulty attending to directions | Yes |
|  | Difficulty dividing attention | Yes |
|  | Distracted | Yes |
|  | Distractible | Yes |
|  | Easily distracted | Yes |
|  | fair | No |
|  | Fair | No |
|  | Fluctuating | Yes |
|  | Follows 1-step commands | No |
|  | Follows one step commands consistently | No |
|  | Follows one step commands with increased time | Yes |
|  | Follows one step commands with repetition | Yes |
|  | Follows one step commands without difficulty | No |
|  | Follows three step commands | No |
|  | Follows two step commands | No |
|  | Good | No |
|  | Impaired | Yes |
|  | Impaired Serial 7s | Yes |
|  | Inattentive | Yes |
|  | Intact | No |
|  | Poor | Yes |
|  | Poor attention/concentration | Yes |
|  | Unable to Concentrate | Yes |
|  | Unable to concentrate | Yes |
|  | Unable to maintain attention | Yes |
|  | WFL | No |
|  | Able to spell WORLD fwd & bkwd | No |
|  | World Incorrect | Yes |
| ***Other cognitive functions*** | Able to problem solve independently | No |
|  | Appropriate decision making | No |
|  | Appropriate for developmental age | No |
|  | Appropriate safety awareness | No |
|  | Assistance required to correct errors made | Yes |
|  | Assistance required to generate solutions | Yes |
|  | Assistance required to identify errors made | Yes |
|  | Assistance required to implement solutions | Yes |
|  | Confused | Yes |
|  | Decreased awareness of errors | Yes |
|  | Decreased command following | Yes |
|  | Does not follow commands | Yes |
|  | Does not require cues | No |
|  | Follows complex commands | No |
|  | Follows commands | No |
|  | Follows all commands and directions without difficulty | No |
|  | Follows all commands without difficulty | No |
|  | Follows commands | No |
|  | Follows multistep commands consistently | No |
|  | Follows multistep commands with increased time | Yes |
|  | Follows multistep commands with repetition | Yes |
|  | Follows multistep commands with repetition | Yes |
|  | Follows multistep commands without difficulty | No |
|  | Follows one step commands | No |
|  | Impaired | Yes |
|  | Impaired decision making | Yes |
|  | Inconsistently follows commands | Yes |
|  | Mild | Yes |
|  | Moderate | Yes |
|  | No command following | Yes |
|  | None | No |
|  | Poor judgement | No |
|  | Poor safety awareness | Yes |
|  | Recognition of people/places | No |
|  | Requires cues for all | Yes |
|  | Requires cues for some | Yes |
|  | Severe | Yes |
|  | Severely Impaired | Yes |
|  | Unable to follow commands | Yes |
|  | WDL | No |
|  | WFL | No |
|  | WNL | No |
| ***Memory*** | 1/3 at 1 minute | Yes |
|  | 1/3 at 3 minutes | Yes |
|  | 2/3 at 1 | Yes |
|  | 2/3 at 3 | Yes |
|  | 3/3 at 1 | No |
|  | 3/3 at 1 & 3/3 at 3 | No |
|  | 3/3 at 3 | No |
|  | 3/3 at 3 minutes | No |
|  | Able to recall 2 of 3 items at 5 mins | Yes |
|  | Able to recall 3 of 3 items at 5 mins | No |
|  | Able to recall date of birth | No |
|  | Appears intact | No |
|  | Confabulation | Yes |
|  | Decreased long term memory | Yes |
|  | Decreased recall of biographical Information | Yes |
|  | Decreased recall of biographical information | Yes |
|  | Decreased recall of precautions | Yes |
|  | Decreased recall of recent events | Yes |
|  | Decreased short term memory | Yes |
|  | Deficit noted | Yes |
|  | Forgetful | Yes |
|  | Grossly Intact | No |
|  | Immediate, recent & remote memory intact | No |
|  | Impaired | Yes |
|  | Impaired Immediate | Yes |
|  | Impaired Long-Term | Yes |
|  | Impaired Recent | Yes |
|  | Impaired Remote | Yes |
|  | Impaired Remote and Recent | Yes |
|  | Impaired Short-Term | Yes |
|  | Impaired/immediate | Yes |
|  | Impaired/recent | Yes |
|  | Impaired/remote | Yes |
|  | Intact | No |
|  | Intact Immediate | No |
|  | Intact Long - term | No |
|  | Intact Recent | No |
|  | Intact Remote | No |
|  | Intact Remote and Recent | No |
|  | Long Term Memory Deficit | Yes |
|  | Memory loss | Yes |
|  | Moderate Memory Dysfunction | Yes |
|  | No short term memory loss | No |
|  | None | No |
|  | Poor Recent | Yes |
|  | Poor recent | Yes |
|  | Poor Remote | Yes |
|  | Poor remote | Yes |
|  | Recalls 3/3 at 3 min | No |
|  | Recalls recent/remote events | No |
|  | Recent - Intact / Remote - Intact | No |
|  | Recent Memory Impaired | Yes |
|  | Recent Memory Intact | No |
|  | Recent/Remote Memory Intact | No |
|  | Remote Memory Impaired | Yes |
|  | Remote Memory Intact | No |
|  | Severe Memory Dysfunction | Yes |
|  | Short Term Memory Deficit | Yes |
|  | Short term memory loss | Yes |
|  | Slight Memory Dysfunction | Yes |
|  | WFL | No |
|  | WNL | No |
| ***Orientation*** | Alert and Oriented in all spheres | No |
|  | Alert and Oriented x 4 | No |
|  | Alert and oriented x 4 | No |
|  | Appropriate for age | No |
|  | Appropriate for developmental age | No |
|  | Date | No |
|  | Disoriented | Yes |
|  | Disoriented to Date | Yes |
|  | Disoriented to person | Yes |
|  | Disoriented to Person | Yes |
|  | Disoriented to place | Yes |
|  | Disoriented to Place | Yes |
|  | Disoriented to time | Yes |
|  | Disoriented to Time | Yes |
|  | Disoriented X4 | Yes |
|  | Has knowledge of person,place,time,and situation | No |
|  | Impaired/person | Yes |
|  | Impaired/place | Yes |
|  | Impaired/time | Yes |
|  | Inconsistent | Yes |
|  | Intact | No |
|  | Intact Oriented x 4 | No |
|  | Intact x 3 person, place, time | No |
|  | Intact x 4 | No |
|  | Knowledge of person,place,time,purpose | No |
|  | No | Yes |
|  | Not Oriented to person | Yes |
|  | Not Oriented to place | Yes |
|  | Not Oriented to time | Yes |
|  | Not Oriented x 4 | Yes |
|  | Orient to Person | No |
|  | Orient to person | No |
|  | Orient to Place | No |
|  | Orient to place | No |
|  | Orient to Situation | No |
|  | Orient to situation | No |
|  | Orient to Time | No |
|  | Orient to time | No |
|  | Orientation to person | No |
|  | Orientation to person, place, and time | No |
|  | Orientation to person, place, time, and situation | No |
|  | Oriented | No |
|  | oriented | No |
|  | Oriented x 4 | No |
|  | Oriented to person | No |
|  | Oriented to Person | No |
|  | Oriented to place | No |
|  | Oriented to time | No |
|  | Oriented x 4 | No |
|  | Oriented X4 | No |
|  | Oriented/disoriented at times | Yes |
|  | Person | No |
|  | Place | No |
|  | Time | No |
|  | Unable to verbalize | Yes |
|  | WDL | No |
|  | WNL | No |
|  | Yes | No |
| *WFL=Within Functional Limits, WNL=Within Normal Limits, WDL=Within Defined Limits.* | | |

***Legend:*** The “Cognitive domain” and “Domain descriptor” labels represent the entries made by the clinician against the cognitive domain heading in the electronic health records. The cognitive deficit column represents the clinical consensus (reached by two psychiatrists, RDG and MT) regarding whether the descriptor refers to a deficit (‘Yes’) or the absence of it (‘No’).

# Online supplemental file, table S4. Results for all cognitive domains and for all cohorts including the whole cohort (referred to as ‘All psychiatric disorders’)

| Population | Comparator | Outcome | Mean ratio  (95% CI) | Mean score with  comparator | Mean score with  semaglutide | p-value |
| --- | --- | --- | --- | --- | --- | --- |
| *All psychiatric disorders* | No treatment | **Cognitive score** | 0.75 (0.65 - 0.85) | 14.91 | 11.14 | 4.1×10^-6^ |
|  | Glipizide |  | 0.83 (0.70 - 0.93) | 13.46 | 11.14 | 0.01 |
|  | Empagliflozin |  | 0.81 (0.69 - 0.94) | 13.69 | 11.14 | 0.004 |
|  | Sitagliptin |  | 0.89 (0.77 - 1.02) | 12.52 | 11.14 | 0.12 |
|  | No treatment | Memory | 0.56 (0.35 - 0.78) | 37.92 | 21.42 | 0.0001 |
|  | Glipizide |  | 0.61 (0.38 - 0.86) | 35.32 | 21.42 | 0.002 |
|  | Empagliflozin |  | 0.56 (0.35 - 0.79) | 38.50 | 21.42 | 0.0003 |
|  | Sitagliptin |  | 0.68 (0.40 - 0.96) | 31.47 | 21.42 | 0.03 |
|  | No treatment | Other cognitive functions | 0.64 (0.51 - 0.80) | 16.17 | 10.37 | 1.3×10^-5^ |
|  | Glipizide |  | 0.69 (0.52 - 0.88) | 15.14 | 10.37 | 0.002 |
|  | Empagliflozin |  | 0.73 (0.56 - 0.94) | 14.21 | 10.37 | 0.01 |
|  | Sitagliptin |  | 0.60 (0.47 - 0.82) | 17.18 | 10.37 | 0.0002 |
|  | No treatment | Attention | 0.83 (0.68 - 1.00) | 17.71 | 14.75 | 0.05 |
|  | Glipizide |  | 0.97 (0.77 - 1.18) | 15.21 | 14.75 | 0.77 |
|  | Empagliflozin |  | 1.04 (0.84 - 1.23) | 14.23 | 14.75 | 0.73 |
|  | Sitagliptin |  | 1.07 (0.85 - 1.30) | 13.75 | 14.75 | 0.54 |
|  | No treatment | Orientation | 0.94 (0.77 - 1.11) | 9.56 | 9.01 | 0.56 |
|  | Glipizide |  | 1.17 (0.92 - 1.44) | 7.68 | 9.01 | 0.20 |
|  | Empagliflozin |  | 0.94 (0.77 - 1.13) | 9.56 | 9.01 | 0.56 |
|  | Sitagliptin |  | 1.10 (0.88 - 1.32) | 8.18 | 9.01 | 0.39 |
| *Major depressive disorder* | No treatment | **Cognitive score** | 0.72 (0.54 - 0.88) | 14.96 | 10.70 | 0.004 |
|  | Glipizide |  | 0.78 (0.58 - 0.97) | 13.75 | 10.70 | 0.05 |
|  | Empagliflozin |  | 0.72 (0.53 - 0.90) | 14.92 | 10.70 | 0.01 |
|  | Sitagliptin |  | 0.84 (0.62 - 1.09) | 12.68 | 10.70 | 0.21 |
|  | No treatment | Memory | 0.60 (0.30 - 0.88) | 39.73 | 23.99 | 0.01 |
|  | Glipizide |  | 0.70 (0.33 - 1.06) | 34.05 | 23.99 | 0.11 |
|  | Empagliflozin |  | 0.61 (0.29 - 0.93) | 39.47 | 23.99 | 0.03 |
|  | Sitagliptin |  | 0.77 (0.36 - 1.21) | 31.00 | 23.99 | 0.28 |
|  | No treatment | Other cognitive functions | 0.68 (0.48 - 0.93) | 15.84 | 10.81 | 0.01 |
|  | Glipizide |  | 0.72 (0.49 - 1.00) | 15.00 | 10.81 | 0.06 |
|  | Empagliflozin |  | 0.72 (0.47 - 0.99) | 15.07 | 10.81 | 0.04 |
|  | Sitagliptin |  | 0.60 (0.41 - 0.88) | 17.95 | 10.81 | 0.002 |
|  | No treatment | Attention | 0.88 (0.65 - 1.17) | 19.41 | 17.00 | 0.35 |
|  | Glipizide |  | 1.05 (0.78 - 1.49) | 16.23 | 17.00 | 0.79 |
|  | Empagliflozin |  | 1.12 (0.83 - 1.62) | 15.15 | 17.00 | 0.50 |
|  | Sitagliptin |  | 1.22 (0.95 - 1.73) | 13.97 | 17.00 | 0.25 |
|  | No treatment | Orientation | 0.96 (0.61 - 1.27) | 8.57 | 8.20 | 0.80 |
|  | Glipizide |  | 1.25 (0.82 - 1.75) | 6.58 | 8.20 | 0.26 |
|  | Empagliflozin |  | 0.83 (0.53 - 1.14) | 9.91 | 8.20 | 0.29 |
|  | Sitagliptin |  | 1.00 (0.66 - 1.30) | 8.23 | 8.20 | 0.98 |
| *Psychotic disorders* | No treatment | **Cognitive score** | 0.77 (0.54 - 1.05) | 23.80 | 18.26 | 0.10 |
|  | Glipizide |  | 0.70 (0.49 - 0.96) | 26.04 | 18.26 | 0.02 |
|  | Empagliflozin |  | 0.85 (0.55 - 1.25) | 21.41 | 18.26 | 0.45 |
|  | Sitagliptin |  | 0.81 (0.54 - 1.19) | 22.42 | 18.26 | 0.29 |
|  | No treatment | Memory | 0.85 (0.47 - 1.21) | 54.25 | 46.06 | 0.41 |
|  | Glipizide |  | 0.83 (0.47 - 1.21) | 55.54 | 46.06 | 0.35 |
|  | Empagliflozin |  | 0.77 (0.43 - 1.31) | 59.56 | 46.06 | 0.30 |
|  | Sitagliptin |  | 0.95 (0.55 - 1.42) | 48.28 | 46.06 | 0.84 |
|  | No treatment | Other cognitive functions | 0.67 (0.37 - 1.13) | 23.85 | 16.05 | 0.09 |
|  | Glipizide |  | 0.72 (0.37 - 1.18) | 22.24 | 16.05 | 0.21 |
|  | Empagliflozin |  | 0.98 (0.47 - 1.65) | 16.37 | 16.05 | 0.95 |
|  | Sitagliptin |  | 0.70 (0.37 - 1.24) | 23.01 | 16.05 | 0.21 |
|  | No treatment | Attention | 0.83 (0.53 - 1.12) | 36.03 | 29.83 | 0.27 |
|  | Glipizide |  | 0.79 (0.51 - 1.07) | 37.62 | 29.83 | 0.17 |
|  | Empagliflozin |  | 1.32 (0.76 - 1.89) | 22.55 | 29.83 | 0.25 |
|  | Sitagliptin |  | 1.05 (0.67 - 1.55) | 28.29 | 29.83 | 0.80 |
|  | No treatment | Orientation | 1.13 (0.67 - 1.75) | 13.18 | 14.84 | 0.67 |
|  | Glipizide |  | 1.15 (0.64 - 1.96) | 12.95 | 14.84 | 0.65 |
|  | Empagliflozin |  | 1.06 (0.54 - 1.82) | 14.04 | 14.84 | 0.86 |
|  | Sitagliptin |  | 1.06 (0.59 - 1.78) | 13.99 | 14.84 | 0.85 |
| *Bipolar disorder* | No treatment | **Cognitive score** | 0.83 (0.56 - 1.10) | 19.19 | 15.95 | 0.23 |
|  | Glipizide |  | 0.77 (0.50 - 1.02) | 20.73 | 15.95 | 0.09 |
|  | Empagliflozin |  | 0.89 (0.59 - 1.22) | 17.86 | 15.95 | 0.53 |
|  | Sitagliptin |  | 0.94 (0.62 - 1.22) | 16.97 | 15.95 | 0.73 |
|  | No treatment | Memory | 0.93 (0.53 - 1.40) | 42.35 | 39.37 | 0.77 |
|  | Glipizide |  | 0.90 (0.51 - 1.35) | 43.92 | 39.37 | 0.65 |
|  | Empagliflozin |  | 0.89 (0.48 - 1.72) | 44.24 | 39.37 | 0.71 |
|  | Sitagliptin |  | 0.97 (0.58 - 1.51) | 40.56 | 39.37 | 0.91 |
|  | No treatment | Other cognitive functions | 0.79 (0.39 - 1.15) | 16.27 | 12.80 | 0.32 |
|  | Glipizide |  | 0.92 (0.47 - 1.90) | 13.89 | 12.80 | 0.80 |
|  | Empagliflozin |  | 0.88 (0.45 - 1.41) | 14.48 | 12.80 | 0.67 |
|  | Sitagliptin |  | 0.65 (0.32 - 1.07) | 19.59 | 12.80 | 0.18 |
|  | No treatment | Attention | 1.15 (0.83 - 1.61) | 32.23 | 37.05 | 0.46 |
|  | Glipizide |  | 1.10 (0.76 - 1.57) | 33.77 | 37.05 | 0.64 |
|  | Empagliflozin |  | 1.74 (1.07 - 2.92) | 21.31 | 37.05 | 0.04 |
|  | Sitagliptin |  | 1.42 (0.96 - 2.05) | 26.01 | 37.05 | 0.11 |
|  | No treatment | Orientation | 0.89 (0.21 - 1.68) | 7.87 | 6.99 | 0.75 |
|  | Glipizide |  | 1.02 (0.25 - 2.07) | 6.83 | 6.99 | 0.95 |
|  | Empagliflozin |  | 0.71 (0.17 - 1.61) | 9.80 | 6.99 | 0.42 |
|  | Sitagliptin |  | 0.97 (0.25 - 1.93) | 7.21 | 6.99 | 0.94 |

***Legend:*** The mean ratio represents the ratio of the mean cognitive score after 12 months of treatment with semaglutide over the mean cognitive score in the comparator treatment strategy.
